# An antibacterial coating based on a polymer/sol-gel hybrid matrix loaded with silver nanoparticles

**DOI:** 10.1186/1556-276X-6-305

**Published:** 2011-04-07

**Authors:** Pedro José Rivero, Aitor Urrutia, Javier Goicoechea, Carlos Ruiz Zamarreño, Francisco Javier Arregui, Ignacio Raúl Matías

**Affiliations:** 1 Nanostructured Optical Devices Laboratory, Electric and Electronic Engineering Department, Public University of Navarra, Edif. Los Tejos, Campus Arrosadía, 31006, Pamplona, Spain

## Abstract

In this work a novel antibacterial surface composed of an organic-inorganic hybrid matrix of tetraorthosilicate and a polyelectrolyte is presented. A precursor solution of tetraethoxysilane (TEOS) and poly(acrylic acid sodium salt) (PAA) was prepared and subsequently thin films were fabricated by the dip-coating technique using glass slides as substrates. This hybrid matrix coating is further loaded with silver nanoparticles using an in situ synthesis route. The morphology and composition of the coatings have been studied using UV-VIS spectroscopy and atomic force microscopy (AFM). Energy dispersive X-ray (EDX) was also used to confirm the presence of the resulting silver nanoparticles within the thin films. Finally the coatings have been tested in bacterial cultures of genus *Lactobacillus plantarum *to observe their antibacterial properties. It has been experimentally demonstrated that these silver loaded organic-inorganic hybrid films have a very good antimicrobial behavior against this type of bacteria.

## Background

Microbes and bacteria are the most abundant of all living organisms in our planet and a large of them are pathogens. Because of that, there is an enormous interest in the research of highly efficient and low cost antibacterial surface treatments and coatings to avoid the apparition of these microorganisms in instrumentals, devices, laboratories, operating rooms, etc. [1,2].

Silver ions show a notorious broad spectrum biocide effect. There are several known mechanisms where the utilization of silver has led to an extraordinary toxicity for bacteria [3-6]. Moreover, silver is particularly attractive because it combines the high toxicity for bacteria with a low toxicity for humans [7-9]. Its disinfectant properties for hygienic and medicinal purposes are known since ancient times, and for example it has been extensively used to prevent wound infection since World War I [10].

Most of the approaches for achieving antibacterial surfaces are based on doping some elements with silver particles which act as silver ion source, for example, in textiles [11,12], surgical instruments [13], and other surfaces [14]. Some authors have reported how silver nanoparticles [15], nanorods [16], or nanotubes [17] are especially efficient antibacterial agents because of their large surface to volume ratio. Up to now such silver nanoparticles have been immobilized on inorganic porous hosts such as zeolites, calcium phosphate, and carbon fiber [18-20]. Moreover, silver-supported silica materials, such as silica glass [21], silica thin films [22], and silica nanoparticles [23], are also good candidates for antibacterial materials due to their fine chemical durability and high antibacterial activity. Moreover, a surface can obtain contact bacteria-killing capacity through chemical modification with tethered bactericidal functionalities such as quaternary amine compounds [24,25], phosphonium salts [26], and titanium oxide particles [27], which are able to kill bacteria upon contact.

However, the biocide efficiency of such coatings depends on the ability of the trapped silver to release ions. Consequently, silver particles with a high specific area show more efficient ion release mechanisms and therefore the antibacterial effect is enhanced. There is a wide variety of coating techniques that have been used for fabricating antibacterial coatings, such as PVD [28], spin-coating [29], or electrospinning [30,31]. In this work, we have developed a facile method to produce an organic-inorganic hybrid matrix by the sol-gel process using the dip-coating technique onto glass substrates. This approach allows to fabricate biocide films in a fast and simple way compared to other fabrication techniques [32].

Polymer-silica hybrid materials have drawn the attention of many researchers recently because of their compatibility with living matter and their promising applications in the medical field [33,34]. Organic polymers in general enjoy a high flexibility, low density, toughness, and easy formability whereas ceramic materials possess other excellent mechanical properties such as a high hardness, combined with a good resistance to high temperature or strong solvents. In this work, these new class of materials are employed to obtain a porous silica surface containing uniformly distributed silver nanoparticles inside the coating. To our knowledge, this is the first time that TEOS/PAA hybrid matrices loaded with silver nanoparticles are fabricated. In addition, their possible antibacterial behavior is also studied.

## Experimental section

### Materials

In this work, the hybrid (organic/inorganic) precursor solution was prepared mixing a water-based solution of poly(acrylic acid sodium salt) (PAA), tetraethoxysilane (TEOS), and ethanol (EtOH). The PAA solution was prepared using ultrapure water (18.2 MΩcm) and its concentration was varied throughout the experiment, from 10^-3 ^to 20·10^-3 ^M respect to the repetitive unit. Silver nanoparticles were further in situ synthesized from silver nitrate and borane dimethylamine complex (DMAB). All chemicals were purchased from Sigma-Aldrich and used without any further purification.

For the bacterial cultures, MRS broth and MRS agar were provided from Fluka. *Lactobacillus plantarum *were obtained from CECT (The Spanish Type Culture Collection University of Valencia). These bacteria are gram-positive, rod, aerotolerant and belong to risk group I.

### Fabrication of the thin films

A starting solution was prepared by mixing together TEOS, EtOH, and the water-based solution of PAA in the following weigh ratio (0.11:0.77:0.12). The pH of the solution was adjusted to 8 by adding NaOH dropwise. The chemicals were mixed under vigorous stirring and the final solution was aged for 30 min. Then the coatings were created by dip-coating. Glass slide substrates were immersed into the starting solution for 15 s and then the substrates were lifted from the solution at a speed of 0.4 mm/s. In order to evaporate very gently the remaining solvents and to allow the consolidation of the coating, samples were stored at room conditions for 3 h. Using this method, high quality transparent coatings were obtained.

Then, the hybrid coating was used as host for in situ silver nanoparticle synthesis. Silver ions were immobilized into the hybrid matrix by ion interchange by simply immersing the coated samples into a AgNO_3 _solution (10 × 10^-3 ^M). During this loading immersion silver cations (Ag^+^) formed electrostatic pairs with some of the carboxylate groups from PAA. This loading step was carried out for 5 min. Afterwards the silver loaded into the coatings were reduced by immersing the samples into a 0.1 M dimethylamine borane (DMAB) solution which act as reducing agent. Therefore the carboxylate-bonded Ag^+ ^ions were reduced to produce zero-valent silver (Ag^0^) particles. Between each loading and reduction step the samples were thoroughly rinsed in ultrapure water. This loading/reduction immersion cycle can be repeated as many times as desired to induce a growth of the silver nanoparticles [35].

### Characterization of the coating film

Atomic force microscopy (AFM) was used to characterize the roughness and the surface morphology of the coating. The samples were scanned using a Veeco Innova AFM, in tapping mode. The optical properties of the antibacterial coatings were characterized by UV-VIS spectroscopy with a Jasco V-630 spectrophotometer. The samples were placed perpendicularly to the light beam during measurement, and a bare glass slide was taken as the reference for the measurements.

EDX spectra were obtained from an INCA X-ray microanalysis system from Oxford Instruments.

### Bacteriologic test method

The bacteriologic tests were carried out using the standard test method [36] which is described in the following paragraphs. The antibacterial coatings were tested in *Lactobacillus plantarum *(CECT # 4005) cultures to observe their antibacterial activities. Their optimal growth conditions are 37°C, 24 h, Tryptic Soy Broth (TSB). *L. plantarum *stock culture was obtained from CECT (The Spanish Type Culture Collection, University of Valencia) and it was maintained by inoculating a loop onto a Tryptic Soy Agar slant and incubating at 37°C for 48 h before storing at 4-10°C.

Test samples were prepared by cutting the coated substrates into 3.5 × 3.5 cm pieces. Three separate pieces of each substrate were prepared for each bacterial strain to be evaluated. Bare glass slides were also cut and prepared following the same procedure. They were disinfected by dipping in 70% isopropyl alcohol and drying in air.

Afterwards, sterile flasks containing TSB were inoculated with the stock culture and incubated for 18 h at 37°C while shaking. From the stock culture 0.2 ml was removed and dispersed in 20 ml of sterile phosphate buffer (50 mM, pH 7.0); vortex well. Using a spectrophotometer zeroed with phosphate buffer at 600 nm, the Optical Density (OD) of the bacterial solution was read. This OD was compared with a previously developed standard curve of OD versus number of viable cells/ml to obtain the approximate number of viable cells. Then the bacterial solution was adjusted by dilution into phosphate buffer to obtain ca. 5 × 105 viable cells/ml. The obtained solution was intended to be exposed to the samples. Additionally, in order to determine the number of viable of organisms in each of the exposure solution, it was made a total of six serial 1:10 dilutions of the cell suspension (10^-1^, 10^-2^, 10^-3^, 10^-4^, 10^-5^, 10^-6 ^dilution), and then it was plated 0.5 ml of the last three dilutions: 10^-4^, 10^-5^, and 10^-6^. They were incubated face down for 24 h at 37°C. After the incubation, it was counted the colony formation units (CFUs) and calculated the CFU/ml in all cases.

Finally, 300 μl of the adjusted bacterial suspension was applied to the plaque sample. Using sterile forceps the bacterial suspension was covered with the coated samples and the bare glass as the control sample, and carefully pressed down to ensure that the liquid spreads to all over the samples, avoiding air bubbles in theirs. Then the samples were introduced into an incubator to >90% relative humidity at 37°C for 24 h.

In order to measure the bacterial killing efficiency of the samples, the remaining bacteria were collected again using the following protocol. The samples were lifted up with forceps and TSB was repeatedly pipetted over the exposed area of the culture medium to suspend as many cells as possible. 0.5 ml of the solution was plated, and three serial 1:10 dilutions, (100, 10^-1^, 10^-2^, 10^-3^) onto TSA plates. Then the inoculated TSA plates were incubated for each sample, face down, at 37°C for 18 h. Finally, after the incubation, the plates was counted for the calculation of the CFU and the CFU/ml.

To measure the effect of an antimicrobial compound, the percentage of cell reduction is calculated between the control sample and the test sample:

A sample is considered biocide if the cell reduction is higher than 99% [36].

## Results and discussion

**Figure 1:**
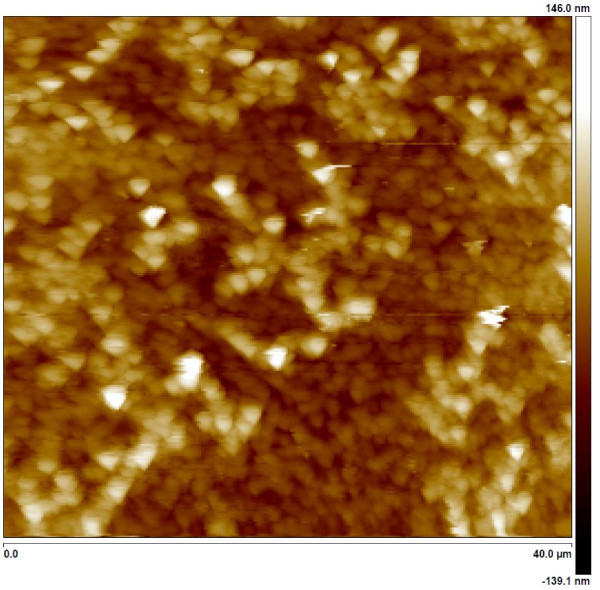
**AFM image topography of one of the samples**. AFM topography of one of the samples (40 × 40 μm).

Firstly the morphology of the thin films was studied by AFM. The resultant coating was uniform and homogeneous, showing a slightly porous surface with an average roughness of 28.3 nm (rms) (Figure 1). It has been reported in Ref. [37] that the basic pH of the TEOS precursor solution gives macroporous aggregates that can be assembled into a film with the dip-coating technique. This matrix shows the advantages of the inorganic materials such as mechanical strength and chemical stability, and at the same time its porosity allows the ion interchange with the external medium, a fundamental aspect when an efficient silver-based antibacterial coating is desired.

Other important aspect of the hybrid matrix is that both organic and inorganic materials should not show any phase separation, in order to get a maximum homogeneity. Other authors have reported how some polymers can interact with metal alkoxides [38], yielding even covalent bonding between the polymers and the inorganic material. In this work the polyelectrolyte PAA was specially selected because under certain conditions, their carboxylic groups could be eventually hydrolyzed and further covalently bonded to tetraorthosilicate particles in a sol-gel process. In a detailed AFM analysis, the phase images of the samples did not show regions with different mechanical stiffness, therefore there were not found any evidence of phase separation in the matrix.

**Figure 2:**
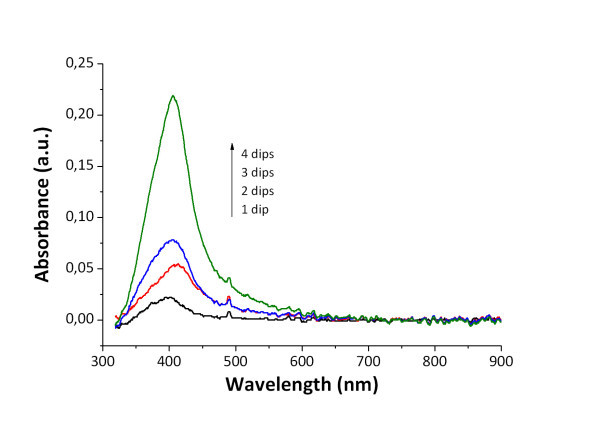
**UV-VIS absorption**. UV-VIS absorption spectra of the coating with different number of loading/reduction.

As it has been commented in the previous section, all the coatings showed a highly transparent appearance immediately after the dip-coating fabrication step. The composition of such coatings consisted of an inorganic matrix with a small proportion of an anionic polyelectrolyte embedded within. Such polymeric chains have carboxylic functional groups that act as host sites for the silver cations during the loading immersion step by ion-interchange. Other authors have reported similar approaches to load polymeric thin films with silver ions [35]. The visual aspect of the hybrid thin films remained unaltered after the silver loading immersion, although the silver ions were already present in the coating. However, the thin films showed a dramatic color change when they were immersed into the DMAB reducing solution. Then the samples turned almost instantaneously into a golden-yellowish color. The visual aspect of the film remained highly transparent and smooth, but with the cited color change. Such alteration of the visible absorption spectrum of the samples change is directly related with the Surface Plasmon Resonance (SPR) phenomenon [39,40] typical of gold and silver nanoparticles. In fact, the UV-VIS absorbance spectrum confirms the existence of an absorption peak near 410 nm (Figure 2).

**Figure 3:**
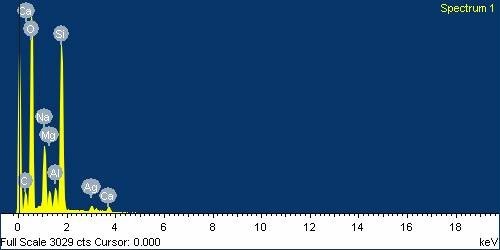
**EDX image**. EDX image of the coating with 4 dip/reduction cycles (PAA 20 mM).

Such narrow absorption bands are due to the SPR phenomenon of the silver nanoparticles synthesized inside the coating. Furthermore, in order to get an additional evidence of the presence of silver within the hybrid coatings an elemental analysis was obtained using the EDX technique (Figure 3). This EDX spectrum shows a peak at 3 keV that confirms the presence of silver within the coating. The rest of the lines of the EDX spectrum correspond to other elements present in the coating (mainly Si, O, and Na) and also present in the glass slide substrate, as far as the coatings were very thin (150 nm approx.). The EDX analysis together with the SPR absorption bands of the UV-VIS spectra make possible to confirm the reduction of silver ions to elemental silver to form nanoparticles inside the organic/inorganic hybrid coating.

One of the most attractive aspects of the approach proposed in this work is that simple variations in the fabrication process can help the designer to tune the overall properties of the coating. In this work two different variations of the initial fabrication process were studied; on one hand the impact of the polyelectrolyte/TEOS ratio, and on the other hand, the number of dip/reduction cycles. The intensity of the SPR absorption band was taken as an indicator of the total amount of synthesized silver nanoparticles.

**Figure 4:**
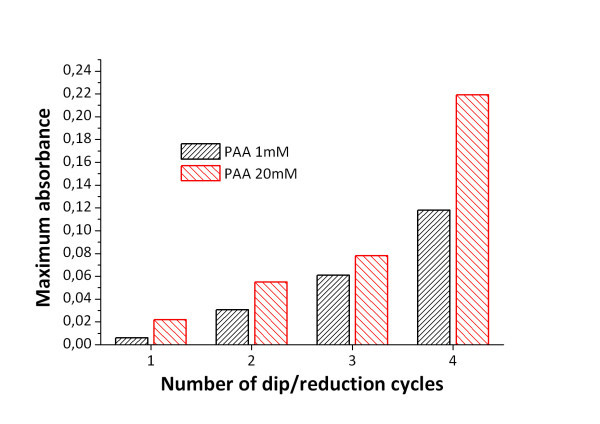
**UV-VIS absorption**. UV-VIS absorption spectra of the coating film with different PAA concentration.

When the samples are immersed into several consecutive loading/reduction cycles the size and amount of the nanoparticles is increased [35]. Figure 2 shows the direct relation between the number of load/reduction cycles and the increasing of the SPR absorption band around 410 nm, proportional to the amount of Ag nanoparticles trapped into the thin film. Moreover, if the concentration of the polymeric solution is varied, the silver synthesis conditions are modified. Figure 4 shows the growth of the intensity of the SPR absorption band as the number of loading/reduction cycles is increased, for two samples with different PAA molar ratio.

The results exposed in Figure 4 confirms the hypothesis that lower polyelectrolyte concentrations results in less host sites (carboxylic functional groups) for the Ag^+ ^cations during the loading step, and after the reduction with DMAB, the amount of silver nanoparticles is significantly lower. When the same loading/reduction protocol was carried out with TEOS only coatings (without polyelectrolyte) significant absorption band was observed in the UV-VIS analysis.

**Figure 5:**
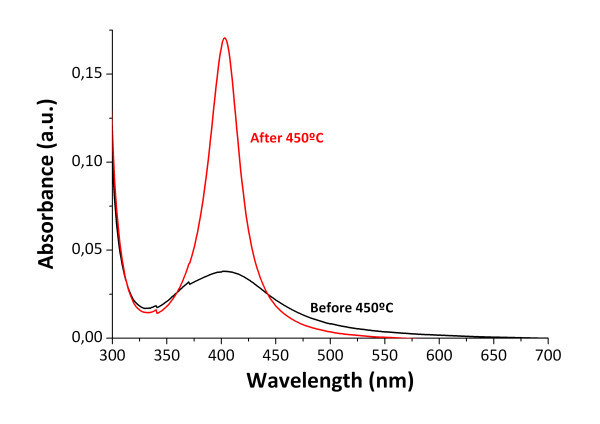
**UV-VIS absorption**. UV-VIS absorbance spectra before and after 450°C thermal treatment.

Moreover, the samples were thermally treated at 450°C for 2 h to increase their mechanical stability. The resulting coatings showed a sharp improvement of the mechanical strength, and a further AFM analysis showed that there was no significant alteration in the morphology of the films. Nevertheless, the UV-VIS absorption spectra of the samples were dramatically modified. After the thermal treatment the SPR absorption peak was significantly narrowed and increased in intensity, as it is shown in Figure 5. This is consistent with the evidences found by other authors in other works [41].

**Figure 6:**
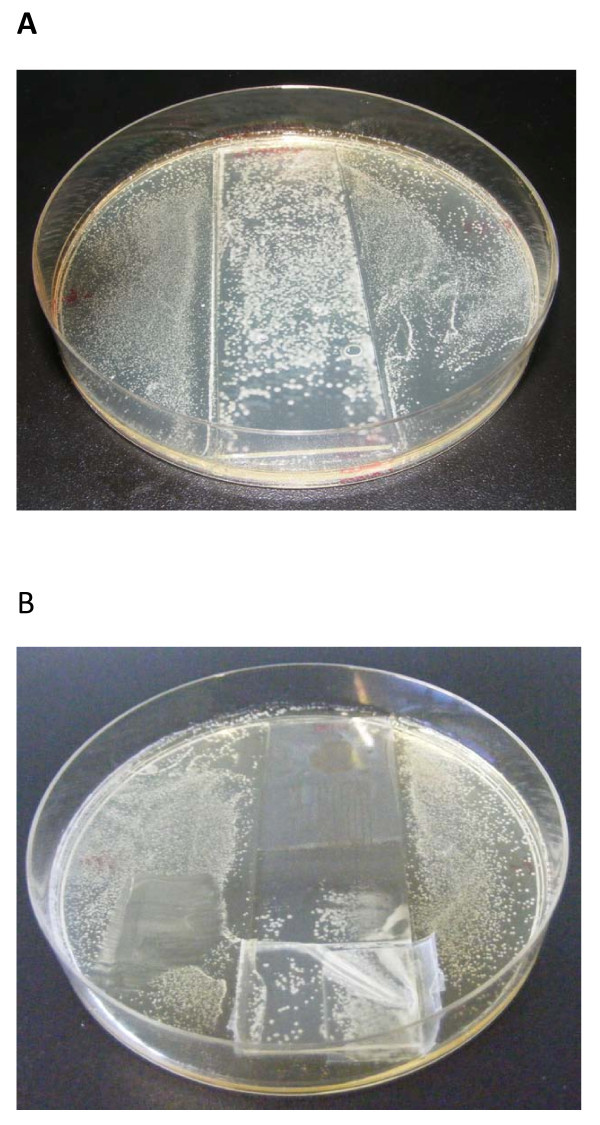
**Bacteria growth**. Bacteria growth on culture plates after 24 h in the **(a) **reference substrate, **(b) **coated substrate. The coated area is clearly visible, as far as it inhibits completely the bacteria growth.

Finally the antibacterial activities against *Lactobacillus plantarum *of the coatings were characterized using the method described in the "Experimental section". Figure 6 shows the results of two samples placed on agar slabs after 24 h. The first one (Figure 6a) shows a reference substrate (a bare glass slide) and it is clearly seen that a high number of *Lactobacillus plantarum *colonies grown up randomly throughout the whole agar slab. The second sample (Figure 6b) has a silver-loaded hybrid coated area where there is no growth of colonies. The behavior is different in the uncoated area of the substrate and in the rest of the agar slab where the growth of colonies is high as in the reference sample (Figure 6a).

All the experiments were performed in triplicate and the treated surfaces reached more than 99.9% of kill efficiency on the growth of *Lactobacillus plantarum*. These results confirm the high antibacterial behavior of the coatings based on silver nanoparticles polymer/sol-gel hybrid matrix.

## Conclusions

In this work, it has been demonstrated that hybrid organic-inorganic coating matrices can be used for in situ silver nanoparticles synthesis, showing excellent antibacterial behavior against *Lactobacillus plantarum*. This approach is a simple and cost-effective method to get coatings with high antibacterial performance, which have most of the advantages of the inorganic coatings, like high mechanical resistance, chemical stability, etc. At the same time, the organic fraction present within the inorganic coating provides the functionality to synthesize the silver nanoparticles that gives the highly efficient antibacterial properties. This gives the ability to tune the overall properties of the film; mechanical, optical, and antibacterial.

The UV-VIS absorbance spectrum confirms the existence of silver nanoparticles inside the coating due to the presence of an absorption peak near 410 nm. Such narrow absorption bands are typical of silver nanoparticles and they are originated by the SPR phenomenon. EDX analysis also confirmed the presence of silver inside the coatings. Moreover, the impact of the organic-inorganic ratio and the number of dip-reduction cycles on the total amount of synthesized silver nanoparticles has been studied. Mechanical resistance of the coatings has been significantly improved using a thermal treatment at 450°C. This process for obtaining antibacterial surfaces can be used for different applications in a wide range of fields like in buildings, pharmaceutical tools, and other instrumental devices.

## Abbreviations

AFM: atomic force microscopy; CFUs: colony formation units; DMAB: dimethylamine borane; EDX: energy dispersive X-ray; PAA: poly(acrylic acid sodium salt); SPR: Surface Plasmon Resonance; TEOS: tetraethoxysilane; TSB: Tryptic Soy Broth.

## Competing interests

I declare that the authors have no competing interests or other interests that might be perceived to influence the results and discussion reported in this paper.

## Authors' contributions

PJR carried out the main part of the experimental work, including the fabrication of the thin films, nanoparticles synthesis, and bacteriological characterization. He participated in the design of the study and in the draft of the manuscript. AU carried the main part of the experimental work, including the fabrication of the thin films, nanoparticles synthesis, and bacteriological characterization. JG participated in the experimental work, carried out the AFM characterization of the films, and contributed with the draft of the manuscript. CRZ participated in the experimental work and carried out the EDX analysis. FJA and IRM participated in the design of the study and helped with the draft of the manuscript.
